# Application of mean-shift clustering to Blood oxygen level dependent functional MRI activation detection

**DOI:** 10.1186/1471-2342-14-6

**Published:** 2014-02-04

**Authors:** Leo Ai, Xin Gao, Jinhu Xiong

**Affiliations:** 1 Department of Biomedical Engineering, University of Iowa, 200 Hawkins Drive C721 GH, Iowa City, IA 52242, USA; 2 Department of Medical Imaging, Suzhou Institute of Biomedical Engineering and Technology, Suzhou, China; 3 Department of Radiology, University of Iowa, Iowa, USA

**Keywords:** Mean-shift, fMRI, BOLD, Clustering

## Abstract

**Background:**

Functional magnetic resonance imaging (fMRI) analysis is commonly done with cross-correlation analysis (CCA) and the General Linear Model (GLM). Both CCA and GLM techniques, however, typically perform calculations on a per-voxel basis and do not consider relationships neighboring voxels may have. Clustered voxel analyses have then been developed to improve fMRI signal detections by taking advantages of relationships of neighboring voxels. Mean-shift clustering (MSC) is another technique which takes into account properties of neighboring voxels and can be considered for enhancing fMRI activation detection.

**Methods:**

This study examines the adoption of MSC to fMRI analysis. MSC was applied to a Statistical Parameter Image generated with the CCA technique on both simulated and real fMRI data. The MSC technique was then compared with CCA and CCA plus cluster analysis. A range of kernel sizes were used to examine how the technique behaves.

**Results:**

Receiver Operating Characteristic curves shows an improvement over CCA and Cluster analysis. False positive rates are lower with the proposed technique. MSC allows the use of a low intensity threshold and also does not require the use of a cluster size threshold, which improves detection of weak activations and highly focused activations.

**Conclusion:**

The proposed technique shows improved activation detection for both simulated and real Blood Oxygen Level Dependent fMRI data. More detailed studies are required to further develop the proposed technique.

## Background

Functional Magnetic Resonance Imaging (fMRI) is a technique that is used to identify regions of activations in the brain. Techniques based on cross-correlation analysis (CCA) and the General Linear Model (GLM) is commonly used for fMRI data analysis [1-7], however these techniques are not without drawbacks. Both techniques typically perform its calculations on a per voxel basis. This means that each calculation does not take into consideration any relationship that neighboring voxels may have with each other. This has the effect of lowering the sensitivity of the technique when looking for activations. This is especially true in low contrast to noise ratio (CNR) situations.

There has been interest in enhancing fMRI data analysis with cluster size tests. Various techniques have been examined with that intention in mind. Cluster analysis (based on random field theory) is commonly used to help isolate activations [8-10]. K-means clustering [11] is a method where observations are partitioned into "k" number of clusters where each observation belongs to the cluster with the closest mean. Fuzzy clustering [12] is similar to k-means clustering, except that fuzzy clustering takes into consideration that a single observation can belong to more than one cluster. Both K-means and Fuzzy clustering have been examined for improving fMRI data analysis [13-16]. Mean-shift clustering (MSC) is another technique to consider for the same purpose.

The MSC technique was first introduced by Fukunaga et al. [17] for examining pattern recognition, but the technique was mostly unexplored until more recently [18,19]. The technique has found uses in image processing and vision tasks. Image segmentation has also been explored with this technique on brain images [20]. MSC revolves around a density estimation that is done on a predetermined feature space. Intuitively, the technique works by calculating the mean shift vector, then shifting the kernel as dictated by the mean shift vector. This process is repeated as appropriate until convergence at which time a cluster in the feature space can be identified. By selectively choosing the features used for the feature space, it would be possible to incorporate characteristics of the data that normally would not be part of the analysis with CCA and GLM based techniques. MSC also offers some other advantages with regards to implementation. The technique does not require assumptions to be made about noise distribution. Compared to typical cluster analysis, no hard cut-off in cluster size is required with MSC. Since MSC is based on density estimation of a feature space, it does not make any assumption on the shape of the clusters either as well as allowing different feature spaces to be used to incorporate different characteristics of the data into analysis. These advantages with MSC may allow higher sensitivity when detecting activations for improved results.

To the best knowledge of the authors, MSC has not been closely explored in the application of fMRI activation analysis. As a first step, this study investigates a straight forward application of MSC to fMRI data analysis.

## Methods

The proposed MSC technique was evaluated using both simulated and real fMRI data. The data analysis was performed using Cross Correlation Analysis (CCA) to generate a statistical parametric image (SPI). The mean-shift clustering was then applied to a feature space constructed using selected characteristics of the SPI. Comparisons were made among CCA, CCA plus cluster analysis (CCA + CA), and CCA + MSC to examine the application of MSC.

### Simulated data

The simulated data was designed to emulate fMRI data using one hundred images, 128 × 128 matrix size, and with a block design of two and a half off/on cycles (20 images per off or on cycle). Activations of various sizes (20 × 20, 10 × 10, 2 × 2 voxels) were inserted onto the data for analysis. Gaussian noise at different CNRs (0.20, 0.40, 0.06, and 0.80) was generated and inserted into the simulated data.

### fMRI data

Ten subjects (5 females, 5 males, age 22–32) gave informed written consent with the approval of the University of Iowa’s (USA) Institutional Review Board. All subjects reported that they were right-handed, not using medications at the time of scanning, healthy, and had no history of any mental or psychiatric conditions. All ten subjects were scanned at the University of Iowa's Medical Education and Research Facility.

Blood Oxygen Level Dependent (BOLD) fMRI data were acquired on a Siemens 3 T Trio scanner (Siemens Medical Solutions, Erlangen, Germany). A gradient echo EPI pulse sequence was used with the following parameters: TR = 2000 ms, flip angle = 90 degrees, TE = 30 ms, matrix = 64 × 64, FOV = 220 mm, slice thickness = 5 mm with 20% gap, 180 images per run. Each scanning session was composed of seven six-minute runs, though only the first run was used for the purpose of this study. A T1 anatomical scan was also performed with the following parameters: TR = 1590 ms, flip angle = 10 degrees, TE = 3.39 ms, matrix = 128 × 128, FOV = 220 mm, slice thickness = 2 mm.

Unilateral electrical stimulation was delivered to the subject’s right median nerve using a Grass S48 stimulator (Grass Technologies, West Warwick, Rhode Island, USA). The stimulation voltage used was 15 volts above the motor threshold, which was individually defined as the minimum voltage required to obtain a thumb twitch. The delivered stimulations were square wave pulses with 0.2 ms duration. A block design of four and a half off/on cycles (40 seconds off, 40 seconds on) with a randomized inter-stimulation interval (ISI) between 1.0-2.0 seconds was used. A randomized ISI was used to reduce any effect that expecting a stimulation occurring with a fixed inter stimulation interval might have on the resulting BOLD signal. The volunteers were asked to passively feel the stimulation, stay still, stay awake, and not actively perform anything else for the duration of the scan.

### Mean shift clustering

MSC is based on density estimation of a predetermined multi-modal feature space of image characteristics. Previously used feature spaces, such as perceived color [21,22], are generally not applicable to fMRI analysis since color is not a feature that would be of interest. For this study, a feature space of the estimated Z values of the SPI and the mean voxel values surrounding a voxel (eight neighboring voxels in 2D and twenty six neighboring voxels in 3D) was used as they can be features of interest and incorporating them in the analysis may help with activation detection. The estimated Z values were used because it relates directly to statistical significance, and the mean voxel value of the surrounding voxels were used to take into consideration neighboring effects. The image features are mapped into a point in a multi-dimension space. The density is calculated within a defined kernel on the feature space. The kernel is moved based on the density gradient in the feature space until the local maximum is found. Points in the feature space associated with the same local maximum are considered to belong to the same cluster, and the calculation is repeated until all points are assigned to a cluster.

Using the Parzen window technique [23], the kernel density estimation at point x can be described by:

(1) f^x=cknhd∑i=1nkx-xih2

where n is the number of data points, *c*_*k*_ is a constant, k is the kernel, h is the kernel size, and d is the number of dimensions in the feature space. The local maximum density is identified at ∇f^x=0 by moving the kernel based on the gradient ascent in the feature space. Equation 1 can be rewritten as:

(2) ∇f^=2cknhd+2∑i=1nx-xik'x-xih2

Assuming g(x) = -k’(x), the gradient density estimator can then be described as:

(3) ∇f^=2cknhd+2∑i=1nxi-xgx-xih2

(4) ∇f^=2cknhd+2∑i=1ngx-xih2×∑i=1nxigx-xih2∑i=1ngx-xih2-x

The first term of Equation 4 is proportional to the density estimate computed with the kernel. The second term:

(5) $$m\left(x\right)=\frac{\overset{n}{\underset{i=1}{\sum }}x_{i}g\left(\frac{x-x_{i}}{h}\right)}{\overset{n}{\underset{i=1}{\sum }}g\left(\frac{x-x_{i}}{h}\right)}-x$$

is the mean shift vector where g is the kernel, h is the kernel size, x is the mean estimate inside the kernel, and x_i_ is the element inside the kernel. The mean shift vector, m(x), defines how the kernel will move along the density gradient towards the local maximum which corresponds with dense regions in the feature space. This calculation is performed at each data point, shifted by m(x) along the density gradient, and repeated until convergence is reached when local maximum is found. This procedure allows the mean shift clustering technique to identify such locations without having to estimate the probability density function of the associated data. Points associated with the same local maximum belong to the same cluster.

Within the mean-shift vector equation, the parameter that likely has the largest effect on the analysis is the kernel size, h, as differences in kernel sizes can change the density estimates calculated which the MSC technique is based on. While adaptive techniques do exist, a range of kernel sizes will be used to examine how the technique will behave.

### Data analysis

The general approach to the proposed MSC analysis method is applying MSC to a feature space constructed using selected characteristics of the SPI generated using CCA. CCA was chosen over GLM because (1) which technique used to generate the SPI is less important for the purposes of this study; (2) the CCA technique allows easy control over the significance level while it is more difficult to do so with GLM.

The real fMRI images were processed using Analysis of Functional NeuroImages (AFNI) [24] and custom Matlab software. As part of the CCA analysis, three-dimensional motion correction was performed to minimize motion effects. All images were normalized to Talairach space ( http://www.bic.mni.mcgill.ca). Constant, linear, and quadratic trends were removed. To investigate the effect of a Gaussian filter on activation detection with MSC, no Gaussian filter and a Gaussian filter with full width half maximum (FWHM) of 4 mm was applied. SPIs were generated for individual subjects.

### Comparisons

The proposed method (CCA + MSC) was compared with typical CCA and CCA plus cluster analysis (CCA + CA) procedure using the same simulated data. Activations of sizes 20 × 20, 10 × 10, and 2 × 2 were inserted onto the data to identify how the techniques behave with different sized activations. The total area of all test patterns were maintained to be the same by varying the number of inserted activations. The 2 × 2 activation size can be considered to be a simulation for highly focused activations. Gaussian white noise was generated and inserted into the simulated data at several CNRs (0.20, 0.40, 0.60, 0.80) for examination on how the technique reacts to noise. No additional smoothing filter was applied to the simulated data.

The proposed technique was assessed based on sensitivity and specificity and compared with the performances of the aforementioned techniques (CCA, CCA + CA, CCA + MSC) on simulated data. True positive rate comparisons were used to examine how the different kernel sizes affect the outcomes at various CNRs, and false positive rate comparisons were used to examine the amount of noise that appear in the results of each technique. Since simulated data was used, the ground truth is known, so identifying true and false positive rates is a simple task by comparing detected activations with true activations. Receiver operating characteristic (ROC) curves were drawn to allow a direct comparison of performance between the techniques.

The real fMRI data from ten subjects was analyzed for evaluating CCA, CCA + CA, and CCA + MSC. The three techniques are evaluated using both individual fMRI data and averaged fMRI data while controlling significance level at p = 0.01. Different statistical thresholds were determined and applied in order to set the significance level to p = 0.01 for comparison purposes. The threshold for CCA was calculated to be z = 4.8 for the filtered and z = 4.9 for the unfiltered (Bonferroni corrected). The CCA + CA used a threshold of z = 2.6 which was calculated based on a study performed by Xiong et al. [10] with a cluster of threshold of 6 voxels for FWHM = 4 mm and 4 voxels for the unfiltered dataset. The CCA + MSC used a threshold of z = 2 for both filtered and unfiltered data. The z threshold for CCA + MSC was selected based on the results of simulations to achieve an approximate significance level of p = 0.01 (see Results section).

## Results

### True positive rate comparison

**Figure 1:**
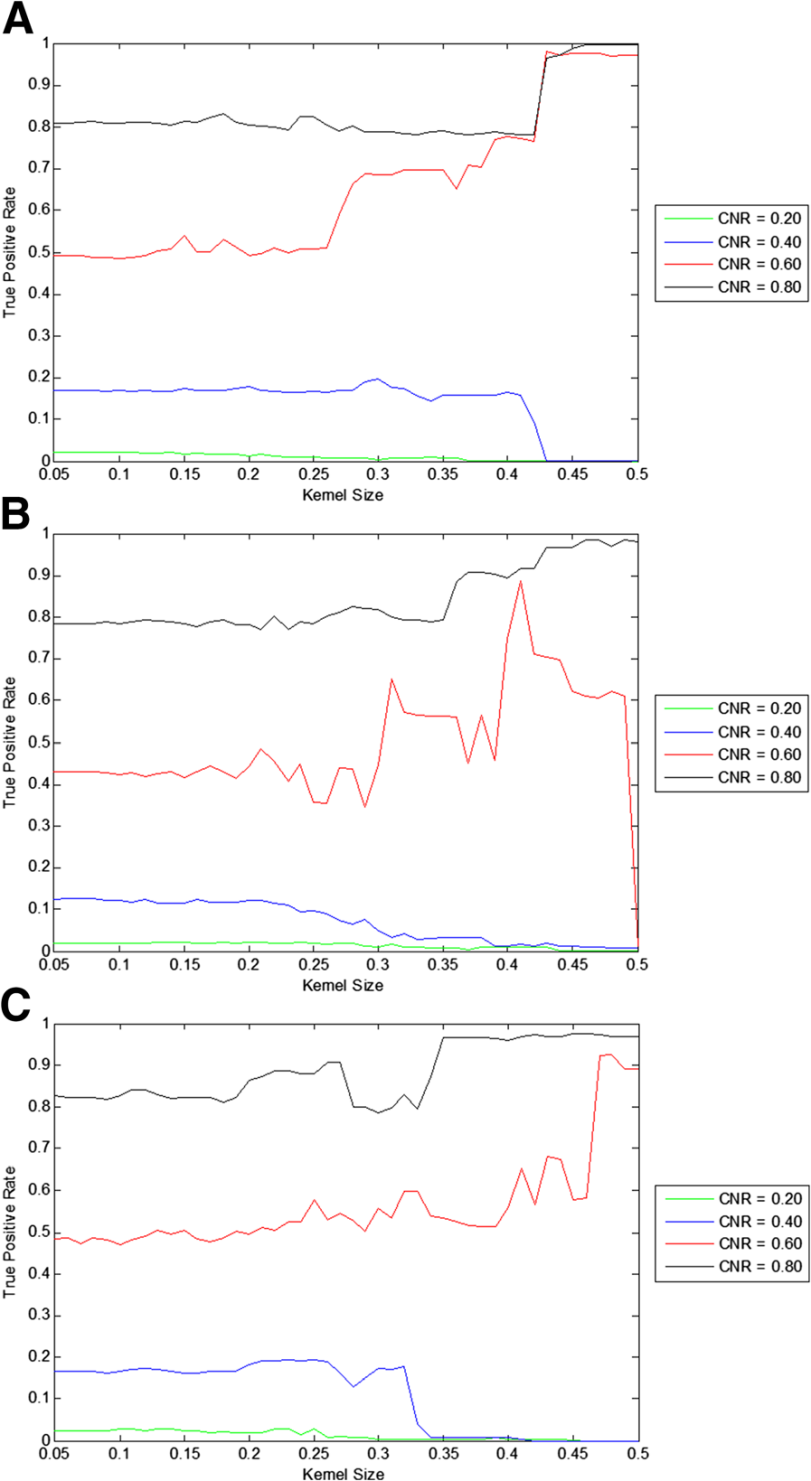
**Effect of kernel size on true positive rates for various activation sizes with simulated data.** The statistical threshold was held constant at z = 3. A range of kernel sizes was used from 0.05 to 0.50. CNRs used are 0.20, 0.40, 0.60, and 0.80. **A**: 20 × 20 activation size. **B**: 10 × 10 activation size. **C**: 2 × 2 activation size.

Using the proposed CCA + MSC technique, true positive rates were plotted to examine the behavior of the technique at various kernel sizes. The statistical threshold was applied at Z = 3. The kernel sizes used were in the range of 0.05 to 0.50. The CNRs of the simulated data are 0.20, 0.40, 0.60, and 0.80. The results show the true positive rate is dependent on the kernel size (Figure 1). For low CNRs (0.20, 0.40), the true positive rates start to decline at a kernel size of 0.25. For very high CNR (0.80), the true positive rate remains high across the range of kernel sizes used. The true positive rates also fluctuates as the kernel size is changed, but this is expected as the noise is being randomly generated and changing the kernel size likely has an effect on the cluster assignment of the voxels. Figure 1 indicates that the kernel size can be optimized for enhanced activation detection. A kernel size of 0.20 was used for the rest of the simulated data comparisons as it seems to be an appropriate choice for all CNR and activation sizes.

### False positive rate comparison

**Figure 2:**
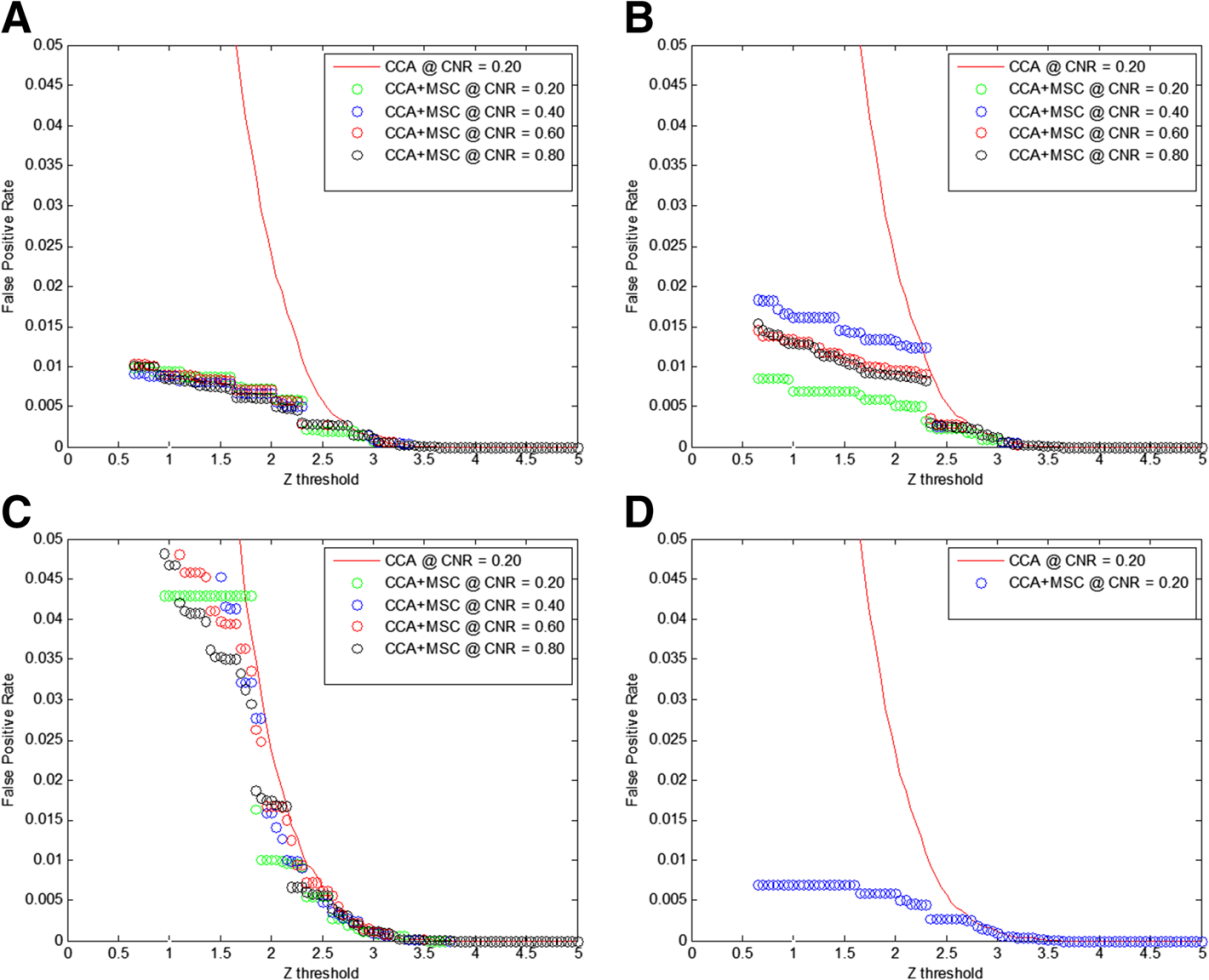
**Change of false positive rates at different z thresholds using various activation sizes with simulated data.** Kernel size was held constant at 0.20. The z thresholds were varied from 0 to 5. CNRs used are 0.20, 0.40, 0.60, and 0.80. **A**: 20 × 20 activation size. **B**: 10 × 10 activation size. **C**: 2 × 2 activation size. **D**: Activation map consisting of noise only.

**Figure 3:**
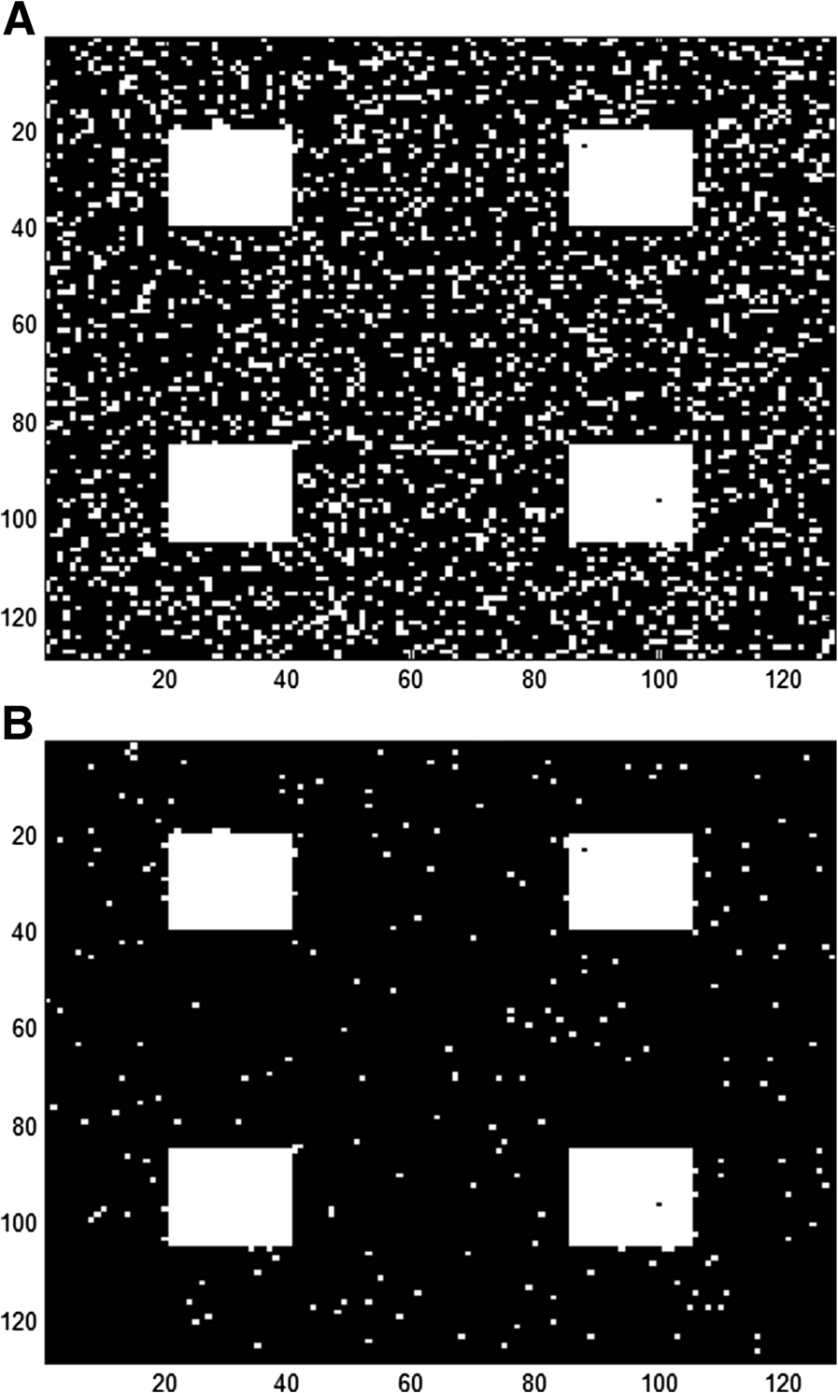
**Activation map of CCA and CCA + MSC.** A threshold of Z = 1 was applied. CNR of 0.80 was used with kernel size of 0.20. **A**: CCA activation map. **B**: CCA + MSC activation map

False positive rates of the proposed CCA + MSC technique were examined while varying the z threshold (0.1 to 5.0) using simulated data. The same CNRs were used. A kernel size of 0.20 was selected based on the true positive rate comparisons (Figure 1). The false positive rate shows significant improvements over CCA at all simulated activation sizes, especially at lower z thresholds (Figure 2). The false positive rate for all activation sizes except 2 × 2 are relatively flat at low z thresholds. It slightly increases as z threshold decreases until z = 0.05 where it increases beyond the figure cap of 0.05 and thus not shown. The 2 × 2 case follows the CCA curve, but an improvement was still seen. The comparison was also made without an activation map (only Gaussian noise), and the result is similar with the 20 × 20 case. Figure 3 shows representative activation maps generated by CCA and CCA + MSC with a z = 1 threshold at CNR = 0.80 with a 0.20 kernel size. It can be visually observed that there are less false activations in the case with CCA + MSC, which agrees with the results from Figure 2.

### ROC comparison of different kernel sizes

**Figure 4:**
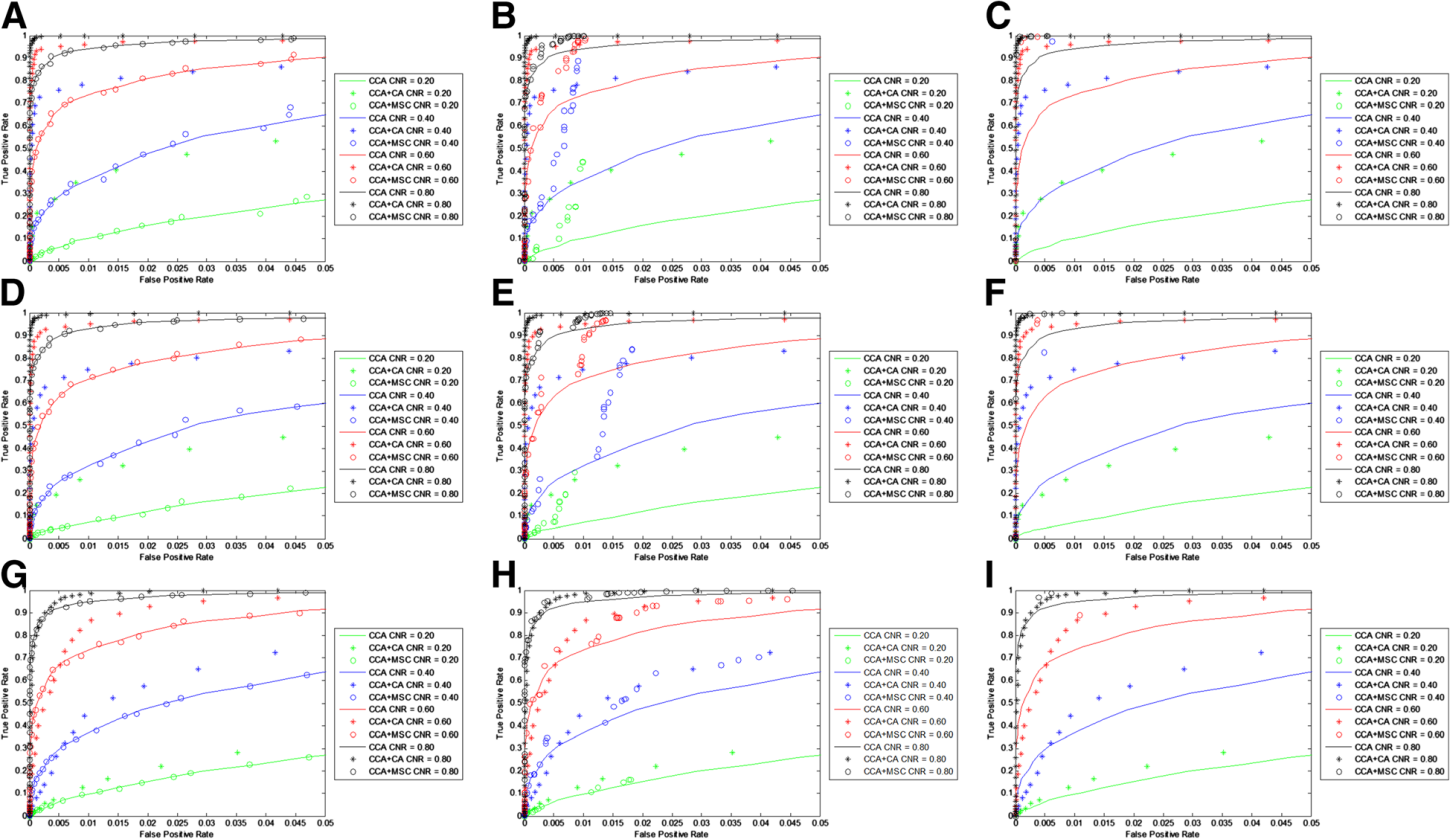
**ROC curves for CCA, CCA + CA, CCA + MSC with different kernel sizes and different activation sizes using simulated data.** Kernel sizes are 0.10, 0.20, and 0.50. Activation sizes used are 20 × 20, 10 × 10, and 2 × 2. CNRs used are 0.20, 0.40, 0.60, and 0.80. **A**: 20 × 20 activation size, kernel size = 0.10 **B**: 20 × 20 activation size, kernel size = 0.20 **C**: 20 × 20 activation size, kernel size = 0.50 **D**: 10 × 10 activation size, kernel size = 0.10 **E**: 10 × 10 activation size, kernel size = 0.20 **F**: 10 × 10 activation size, kernel size = 0.50 **G**: 2 × 2 activation size, kernel size = 0.10 **H**: 2 × 2 activation size, kernel size = 0.20 **I**: 2 × 2 activation size, kernel size = 0.50.

A comparison was made with ROC curves between CCA, CCA + CA, and the proposed CCA + MSC at the same CNRs as before, and on the same simulated data set (Figure 4). Simulated data was used for this comparison with the statistical threshold varied from Z = 0.1 to 5.0 and the kernel size being set at 0.10, 0.20, and 0.50. The ROC curves indicate that a properly chosen kernel size, in this case 0.20, show an improvement over both CCA and CCA + CA. If the kernel size (e.g., 0.50) used is too large, essentially no activations are detected using CCA + MSC. If the kernel size (e.g., 0.10) is too small, the CCA + MSC technique is inferior to CCA + CA and performs similarly with CCA. This would be consistent with the true positive rate comparison (Figure 1).

The relative performance of CCA + MSC and CCA + CA shows a rather complex relationship. In the 20 × 20 and 10 × 10 cases at kernel size = 0.20, CCA + MSC is better than CCA + CA when the false positive rates range from 0.01 and 0.05. It is inferior to CCA + CA when the false positive rate is below 0.01. Considering that significance levels of p = 0.05 and p = 0.01 are commonly used for activation detection, CCA + MSC should show improvement over CCA + CA in a practical situation. In the 2 × 2 case, CCA + MSC appears to be better than CCA + CA up to a false positive rate of about 0.01, then becomes worse until about 0.02, and the two techniques perform similarly after that.

### CCA vs CCA + MSC vs CCA + CA on real data

**Figure 5:**
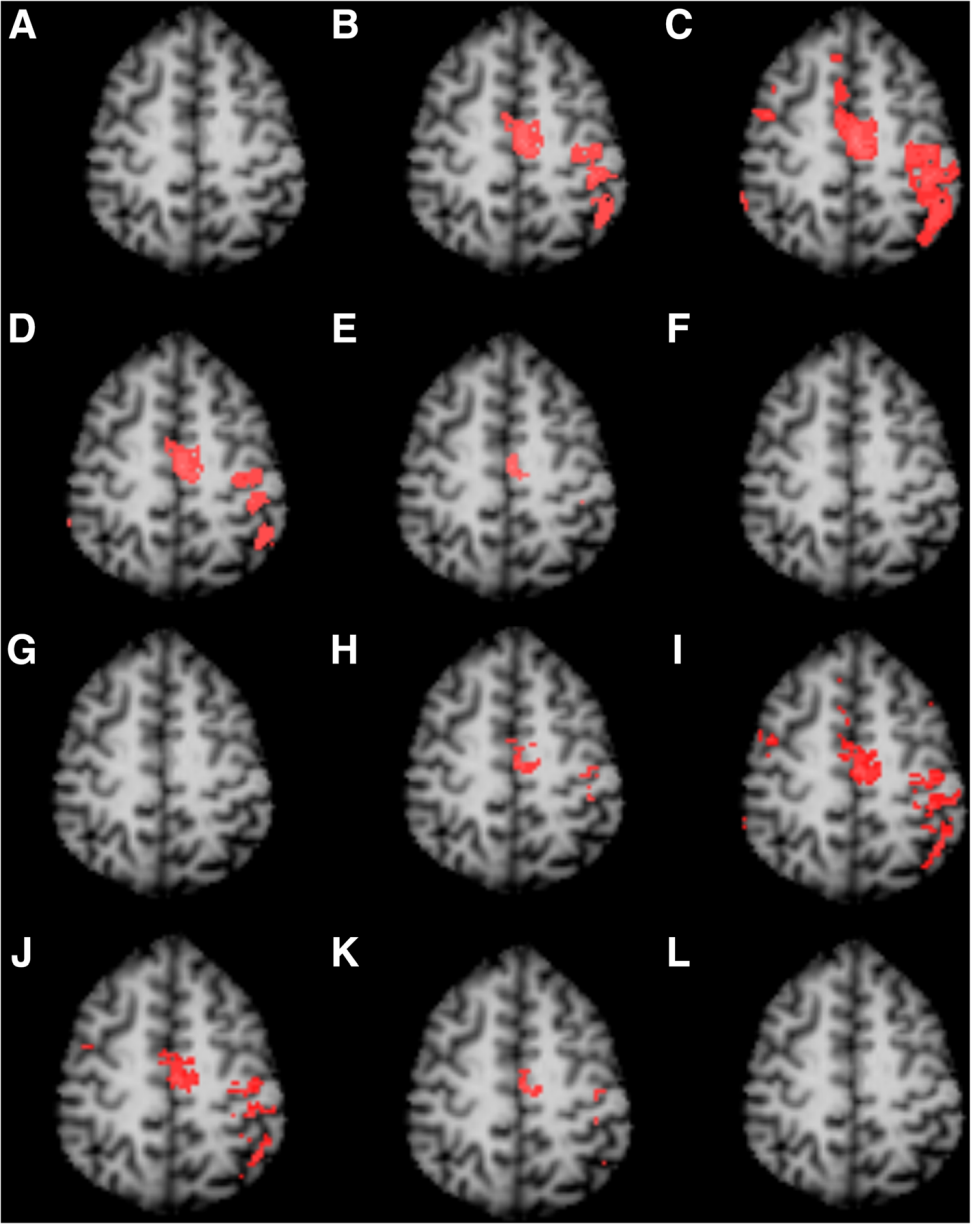
**Activation of median nerve stimulation detected with CCA, CCA + CA, CCA + MSC.** Significance levels were controlled at p = 0.01 for all images. Z thresholds were changed for each technique based on the significance level. **A**: CCA, Z = 4.8, FWHM = 4 mm **B**: CCA + CA, Z = 2.6, cluster size threshold = 6 voxels, FWHM = 4 mm **C**: CCA + MSC, Z = 2, kernel size = 0.05, FWHM = 4 mm **D**: CCA + MSC, Z = 2, kernel size = 0.10, FWHM = 4 mm **E**: CCA + MSC, Z = 2, kernel size = 0.15, FWHM = 4 mm **F**: CCA + MSC, Z = 2, kernel size = 0.20, FWHM = 4 mm **G**: CCA, Z = 4.8, no filter applied **H**: CCA + CA, Z = 2.6, cluster size threshold = 4 voxels, no filter applied **I**: CCA + MSC, Z = 2, kernel size = 0.05, no filter applied **J**: CCA + MSC, Z = 2, kernel size = 0.10, no filter applied **K**: CCA + MSC, Z = 2, kernel size = 0.15, no filter applied **L**: CCA + MSC, Z = 2, kernel size = 0.20, no filter applied.

The real fMRI data was analyzed using CCA, CCA + CA, and CCA + MSC (Figure 5). Activations are expected to be seen on the left M1/S1 region due to the right median nerve stimulation. The CCA + MSC was applied with different kernel sizes of 0.05, 0.10, 0.15, and 0.20. Based on Figure 2, a threshold of z = 2 was applied to achieve a significance of p = 0.01. As shown in Figure 4, the expected activations can be detected using CCA + CA and CCA + MSC with or without a filter in the expected areas while no activation can be seen with standard CCA. Table 1 summarizes the activation volume and average z-scores of the averaged data of both filtered and unfiltered fMRI data. The optimal kernel size for real fMRI is 0.05 and 0.10, which is smaller than the kernel size of 0.20 for the simulated data. Both kernel sizes of 0.05 and 0.10 cases show activations with the 0.10 case showing slightly smaller activations than CCA + CA and the 0.05 case showing larger activations when compared to CCA + CA. In the cases with no filter applied, the detected activations are smaller than their filtered counterparts (Table 1) likely due to the filter enhancing CNR of the SPI, but the non-filtered results generally show the same trends as the filtered results. Individual results are summarized in Table 2 and essentially follow the same trends seen in Table 1. The results show that CCA, CCA + CA, and CCA + MSC (kernel size = 0.10) are statistically different when unfiltered (ANOVA, F = 15.4, p < 0.05) or filtered (ANOVA, F = 10.9, p < 0.05). The Tukey test further reveals that CCA + MSC is significantly better than CCA in both filtered and unfiltered cases (p = 0.05). The performance of CCA + MSC is statistically similar with CCA + CA (p = 0.05).

**Table 1:** Activation volume and average Z-scores for averaged real fMRI data

	**FWHM = 0 mm**		**FWHM = 4 mm**	
	**Volume**	**Z mean**	**Volume**	**Z mean**
CCA	0	0.0	0	0.0
CCA + CA	18	3.1	134	3.1
CCA + MSC 0.05	127	2.4	311	2.6
CCA + MSC 0.10	64	2.5	109	3.1
CCA + MSC 0.15	6	3.0	1	4.0
CCA + MSC 0.20	0	0.0	0	0.0

Note: Volumes are measured in voxels.

**Table 2:** Activation volume and average Z-score for individual subjects on real fMRI data

	**FWHM = 0 mm**						**FWHM = 4 mm**					
	**CCA**		**CCA + CA**		**CCA + MSC**		**CCA**		**CCA + CA**		**CCA + MSC**	
**Subject**	**Volume**	**Z mean**	**Volume**	**Z mean**	**Volume**	**Z mean**	**Volume**	**Z mean**	**Volume**	**Z mean**	**Volume**	**Z mean**
1	45	7.5	163	5.3	212	6.4	128	7.4	200	6.1	200	6.4
2	20	6.1	114	4.0	169	3.6	18	6.8	106	3.7	181	3.8
3	15	5.8	106	3.9	136	3.5	18	5.8	181	3.6	258	3.2
4	0	0.0	58	3.3	98	2.9	0	0.0	23	3.4	135	2.9
5	102	6.4	251	4.9	265	4.9	182	6.4	390	5.1	395	5.1
6	78	6.6	159	5.2	165	5.1	137	6.7	285	5.2	301	5.1
7	30	6.4	135	4.8	146	4.7	51	6.4	192	4.8	243	5.0
8	0	0.0	41	3.6	60	3.2	6	5.3	64	3.6	88	3.1
9	31	6.3	66	5.0	70	4.9	60	6.3	181	4.6	180	4.5
10	239	8.4	292	7.7	299	7.6	402	9.0	424	8.7	423	8.7
Mean	55.9	5.4	138.5	4.8	162.0	4.7	100.2	6.0	204.6	4.9	240.4	4.8
Standard deviation	72.0	2.9	81.8	1.2	78	1.5	123.1	2.3	130.3	1.6	107.7	1.8

Note: Volumes are measured in voxels.

## Discussion

In this study, the adoption of MSC into fMRI analysis was examined by comparing it to CCA and CCA + CA. The ROC curves (Figure 4) indicate that the proposed MSC technique show an improvement over CCA and an improvement over CCA + CA when the false positive rate is above 0.01 in most cases. The false positive rate comparisons (Figure 2) showed significant improvement over CCA which indicates that CCA + MSC controls noise very well. This allows a lower statistical threshold to be used when identifying activations (Figure 3). Another potential benefit of MSC is for highly focused activation detection since no cluster threshold is applied.

The performance of CCA + MSC depends on the kernel size being used (Figure 1), but determining an optimal range of kernel sizes is not a trivial issue. If the kernel size used is too large, no activations would be detected. Conversely, if the kernel size used is too small, the proposed technique does not show an improvement when compared to CCA and CCA + CA. A proper kernel size needs to be used in order to see an improvement over CCA and CCA + CA. When tested with simulated data, the ROC curves and true positive comparison indicate that a kernel size of roughly 0.20 should show an improvement over CCA (Figure 4), but no activations were detected with real fMRI data if that kernel size is used. Real fMRI data showed activations at kernel size of 0.05 and 0.10 (Figure 5). The difference in optimal kernel sizes between the real and simulated data may be explained by the different noise characteristics of the data. The simulated data is the ideal situation with only Gaussian white noise. Real fMRI data will have multiple noise types such as movement artifacts, physiological noise, noise from the MRI machine itself, etc. It is likely that the optimal kernel size depends on the structure of noise in the data and needs optimized for each individual data set. Future studies are required to examine this issue in more detail.

Significant improvement is seen with CCA + MSC over CCA on the simulated data in the ROC curves in the 20 × 20 and 10 × 10 cases at a kernel size of 0.20 (Figure 4B and E), but this improvement is sudden and is concentrated in the region of false positive rates up to 0.02. While this type of improvement is typically unexpected, it is consistent with Figure 2 where false positive rates are shown to be well controlled (lower than 0.02) at a large range of z thresholds for all cases except the 2 × 2 case. This explains why the data is concentrated in the region of false positive rate up to 0.02.

The false positive rate comparison for the 2 × 2 test pattern showed a curve that is similar to the CCA curve, which does not follow the 10 × 10 and 20 × 20 cases (Figure 2). This may be due to the 2 × 2 case having many more neighboring voxels adjacent the test pattern than the other cases. Four 20 × 20 test patterns were inserted into simulated fMRI images which results in 320 neighboring voxels. To maintain the same total area of the test pattern, four hundred 2 × 2 activations were used, resulting in 3200 neighboring voxels. The 2 × 2 test image has ten times the number of voxels that are directly adjacent the inserted activations when compared to the 20 × 20 test image. It is more likely for falsely activated voxels to be detected by cluster analysis techniques if it is attached to the test pattern than when isolated. More falsely activated voxels are expected to be detected for the 2 × 2 test pattern, thus increasing the false positive rate. Regardless, the proposed technique still shows improvement when compared to CCA in the 2 × 2 case.

CCA + MSC can show an improvement over CCA + CA at a false positive rate of greater than about 0.01 with a kernel size of 0.20 (Figure 4), but this improvement is not seen in the 2 × 2 case where CCA + CA was either superior or about the same as CCA + MSC. A cluster threshold of 4 voxels was used to set the significance value at p = 0.01, which also happens to be the exact size of the simulated activations in the 2 × 2 case. If the activation size was three voxels or a different significance level was used and the voxel cluster threshold was increased, the activations would have been removed by the cluster analysis. The CCA + MSC does not have a cluster threshold and has the potential of better detecting highly focused activations.

The selection of image characteristics used in the feature space should be examined in future studies as the feature space will likely have a large effect on the results as well as the range of acceptable kernel sizes. The image characteristics selected for the featured space used in this study was the estimated Z values found in the SPI and the mean voxel values surrounding a voxel. While there are many methods of constructing a feature space, the feature space used did show an improvement over CCA in the simulated and real fMRI data at the same significance level, it is unknown what image features or what combination of image feature will produce the best feature space for fMRI analysis. The feature space used in this study does not incorporate temporal features in the data or positional features for example. There may be other types and combinations of image features that can be used, and it would certainly be an area of further examination.

The proposed technique has the limitation that the significance levels cannot be easily theoretically calculated (or at least we have not been able to come up with a method of doing so). This presents a particular drawback when doing comparisons. Significance levels can be approximated using simulations as was done in this study, but still may present some challenges when very accurate comparisons are required.

## Conclusion

The experiment performed in this study examines the application of MSC to CCA in fMRI activation detection. The results show that an improvement with CCA + MSC can be seen over the typical CCA and CCA + CA analysis technique. The proposed technique maintains a low false positive rate which allows the use of lower statistical thresholds while controlling for noise and helps activation detection in low CNR situations. This also helps in detecting small highly focused activations especially considering that CCA + MSC does not require the application of a cluster size threshold, which is required by most cluster analysis techniques. By nature, CCA + MSC also has the ability to incorporate different image characteristics into a feature space for analysis. These benefits can help to improve activation detection in fMRI data. However, studies in the optimization in kernel size and feature space are needed to further develop the proposed technique. Despite the aforementioned limitations, the proposed technique shows promise in improving fMRI activation detection.

## Abbreviations

BOLD: Blood oxygen level dependent; CCA: Cross correlation analysis; CNR: Contrast to noise ratio; fMRI: Functional magnetic resonance imaging; GLM: General linear model; MSC: Mean shift clustering; ROC: Receiver operating characteristic; SPI: Statistical parametric image.

## Competing interests

None of the authors have any conflict of interest or competing interests with regard to the findings presented in this manuscript, financial, or otherwise.

## Authors’ contributions

Study design; LA, GX, JX; Data collection: LA, JX; Analysis; LA, JX; Interpretation; LA, GX, JX. All authors read and approved the final manuscript.

## Pre-publication history

The pre-publication history for this paper can be accessed here:

http://www.biomedcentral.com/1471-2342/14/6/prepub
